# Seasonal malaria chemoprevention in Africa and China’s upgraded role as a contributor: a scoping review

**DOI:** 10.1186/s40249-023-01115-x

**Published:** 2023-07-05

**Authors:** Ming Xu, Yun-Xuan Hu, Shen-Ning Lu, Muhammad Abdullahi Idris, Shu-Duo Zhou, Jian Yang, Xiang-Ning Feng, Yang-Mu Huang, Xian Xu, Ying Chen, Duo-Quan Wang

**Affiliations:** 1 Department of Global Health, School of Public Health, Peking University, Haidian District, 38 Xue Yuan Road, Beijing, 100191 China; 2 Institute for Global Health and Development, Peking University, Beijing, China; 3 National Institute of Parasitic Diseases, Chinese Center for Disease Control and Prevention (Chinese Center for Tropical Diseases Research), NHC Key Laboratory of Parasite and Vector Biology, WHO Collaborating Centre for Tropical Diseases, National Center for International Research On Tropical Diseases, Shanghai, China; 4 Physiotherapy Department Federal Medical Center, Zamfara State Gusau, Nigeria; 5 Anhui Provincial Center for Disease Control and Prevention, Hefei, China; 6 School of Global Health, Chinese Center for Tropical Diseases Research, Shanghai Jiao Tong University School of Medicine, Shanghai, China

**Keywords:** Seasonal malaria chemoprevention, Malaria, Prevention, China’s contribution, Multilateral partnership

## Abstract

**Background:**

Children under five are the vulnerable population most at risk of being infected with *Plasmodium* parasites, especially in the Sahel region. Seasonal malaria chemoprevention (SMC) recommended by World Health Organization (WHO), has proven to be a highly effective intervention to prevent malaria. Given more deaths reported during the COVID-19 pandemic than in previous years due to the disruptions to essential medical services, it is, therefore, necessary to seek a more coordinated and integrated approach to increasing the pace, coverage and resilience of SMC. Towards this end, fully leverage the resources of major players in the global fight against malaria, such as China could accelerate the SMC process in Africa.

**Methods:**

We searched PubMed, MEDLINE, Web of Science, and Embase for research articles and the Institutional Repository for Information Sharing of WHO for reports on SMC. We used gap analysis to investigate the challenges and gaps of SMC since COVID-19. Through the above methods to explore China’s prospective contribution to SMC.

**Results:**

A total of 68 research articles and reports were found. Through gap analysis, we found that despite the delays in the SMC campaign, 11.8 million children received SMC in 2020. However, there remained some challenges: (1) a shortage of fully covered monthly courses; (2) lack of adherence to the second and third doses of amodiaquine; (3) four courses of SMC are not sufficient to cover the entire malaria transmission season in areas where the peak transmission lasts longer; (4) additional interventions are needed to consolidate SMC efforts. China was certified malaria-free by WHO in 2021, and its experience and expertise in malaria elimination can be shared with high-burden countries. With the potential to join the multilateral cooperation in SMC, including the supply of quality-assured health commodities, know-how transfer and experience sharing, China is expected to contribute to the ongoing scale-up of SMC.

**Conclusions:**

A combination of necessary preventive and curative activities may prove beneficial both for targeted populations and for health system strengthening in the long run. More actions are entailed to promote the partnership and China can be one of the main contributors with various roles.

## Background

**Fig. 1:**
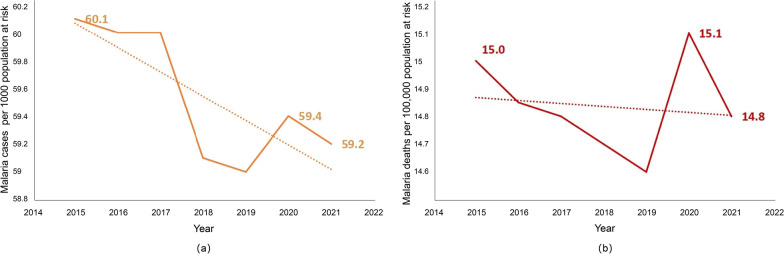
Global trends in malaria case incidence and mortality rate; **a** Malaria cases per 1000 population at risk; **b** Malaria deaths per 100,000 population at risk; Source: World Health Organization estimate

Malaria is typically transmitted to humans by the bite of a female *Anopheles* mosquito, the carrier of Plasmodium parasites [1]. Malaria outbreaks have been linked to climatic anomalies associated with the El Niño-Southern Oscillation phenomenon [2]. During historical El Niño events (inter annual time scale), the timing of malaria outbreaks did not change from the annual cycle, but the number of cases intensified [3]. The burden of malaria remains severe on the African continent, and it was reported by World Health Organization (WHO) that malaria cases had increased from 218 million in 2020 to 232 million in 2021, and the number of deaths had increased from 544,000 to 590,000. Global trends in malaria case incidence and mortality rate are shown in Fig. 1. According to World Malaria Report 2022, there were an estimated 247 million malaria cases in 2021 in 84 malaria-endemic countries (including the territory of French Guiana), an increase from 245 million in 2020, with most of this increase coming from countries in the WHO African Region, and estimated deaths declined slightly in 2021 to 619,000. The coronavirus disease 2019 (COVID-19) pandemic has negatively impacted the prevention of malaria control. With lockdowns in many countries, supply chains of health commodities were cut short. Malaria funding was below what was required to achieve global goals, and many countries faced competing health priorities in the context of severely constrained resources. Although COVID-19 was less often severe in children than in older people, this group would bear a disproportionate burden of excess malaria mortality from COVID-19-related disruption of health systems and malaria control programs [4]. Between 2019 and 2021, 63,000 deaths were due to disruptions to essential malaria services during the COVID-19 pandemic [5]. The vast majority of malaria deaths occurred among children under 5 years of age, especially in the Sahel region, which is over 3800 km long between the Sahara Desert in northern Africa and the grasslands of central Sudan, spanning ten countries including Senegal, Mauritania, Mali, Burkina Faso, Niger, Nigeria, Chad, the Republic of Sudan, the Republic of South Sudan, and Eritrea.

There are multiple interventions to prevent malaria infection among children. Seasonal malaria chemoprevention (SMC) is an ideal choice for environments with strong seasonal transmissions. For settings with moderate-to-high perennial or seasonal transmission, intermittent preventive treatment of malaria in school-aged children (IPTsc) can also be adopted for children aged 5–15 years but its introduction should not compromise chemoprevention interventions for children under 5 years of age, who are at highest risk of severe malaria. Post-discharge malaria chemoprevention (PDMC) can be chosen for children with severe anemia after they are discharged from a hospital.

SMC is the intermittent administration of complete courses of antimalarial medicines during the malaria season: a combination of sulfadoxine-pyrimethamine (SP) and amodiaquine (AQ). SMC is a highly effective intervention to prevent malaria infection during the peak transmission period among the vulnerable population most at risk: children under five. Several studies have also proved its safety [6]. In 2012, WHO recommended SMC as a malaria prevention strategy for children 3–59 months [7]. The latest progress shows that the role of mothers who can lead their children to take SP and AQ plays a significant role in promoting SMC in Nigeria. A study in Burkina Faso shows that it is also essential to treat and screen the families of children who use SMC simultaneously.

United Nations International Children's Emergency Fund (UNICEF), the Global Fund to Fight AIDS, Tuberculosis and Malaria, the World Bank, United, and the US President’s Malaria Initiative are major partners that invest and program work in concert with the WHO malaria programme, the Malaria Consortium, West Africa Health Organization, and related organizations to promote SMC. In late 2020, the Korean International Cooperation Agency (KOICA) joined forces to fund the Expanding System Capacity and Availability of Malaria Chemoprevention in the Sahel Region programme (SMC IMPACT). To fully implement SMC within one year of four cycles, about 4 USD will be needed to protect a child. The average cost for large-scale implementation is 3.38 USD/child per year and 4.27 USD/child per year for small-scale implementation. Gilmartin et al. analyzed the financial costs and actual benefits of SMC implementation in seven countries in the Sahel region. The conclusion strongly indicates that SMC investment has achieved high returns at a meager cost, which is worth popularizing [8].

The study’s main objective is to seek a more coordinated and integrated approach to increasing the pace and coverage of SMC. More importantly, to explore the opportunities to borrow the key learning from China, given its long-term cooperation with African countries in rolling back malaria through the sharing of expertise and provision of quality-assured antimalarials and Long Lasting Insecticidal Mosquito Nets (LLINs). An expanded alliance with a broader engagement of stakeholders will be conducive to achieving the goal of SMC and stepping up the fight to eliminate malaria in Africa.

## Methods

### Search strategy

A search was performed for research articles from January 1, 2019 to January 31, 2023 through the electronic databases of PubMed, MEDLINE, Web of Science, and Embase. The search was focused on: (1) the development of SMC since the outbreak of the COVID-19 pandemic; (2) challenges SMC has faced since the outbreak of the COVID-19 pandemic; (3) SMC’s current gaps and trends of the development; (4) China’s contribution to SMC given the constructive consultations and cooperation between Chinese Center for Disease Control and Prevention (CDC) and key partners in SMC in recent years. To maintain the search comprehensiveness, we searched research articles with the following terms in their titles, abstracts, keywords, or topic: “seasonal malaria chemoprevention”, “malaria”, “development” or “progress”, “challenge”, “gap” or “disparity”, “China” or “Chinese”. We conducted an additional search in Institutional Repository for Information Sharing of WHO in the past 20 years. Inclusion criteria: (1) Being pertinent to the subject of SMC; (2) There was no restriction on the type of research article; (3) No restrictions were made on languages for the article search; (4) What have been published in national and international peer-reviewed journals or reports from relevant organizations. Exclusion criteria: (1) Being irrelevant to the subject of SMC, such as studies that did not use SP and AQ as the exposure; (2) Duplicate research articles; (3) Grey article reports.

### Gap analysis

Gap analysis of SMC was conducted from the four fields: development, challenges, gaps of SMC projects since the outbreak of the COVID-19 epidemic, and China’s contribution to SMC. We additionally collected data from a Chinese medicine industry named Guilin Pharma, which has produced dispersible SPAQ-CO (combined packaging of sulfadoxine-pyrimethamine and amodiaquine) for SMC, including the doses of dispersible SPAQ-CO exported to relevant countries and clients in the past 3 years.

### Interview with the major manufacturer to provide antimalarials for SMC

An interview was conducted with a concerned staff of the Fosun Pharma management team about the supply of antimalarials to SMC on November 8, 2022. Fosun Pharma is a major supplier of antimalarials for SMC. Its WHO-prequalified dispersible SPAQ-CO has been widely used in the Sahel region and ranks first in terms of the volume provided for children under five years old, both through multilateral and bilateral channels [9].

## Results

### Characteristics of research articles included

**Fig. 2:**
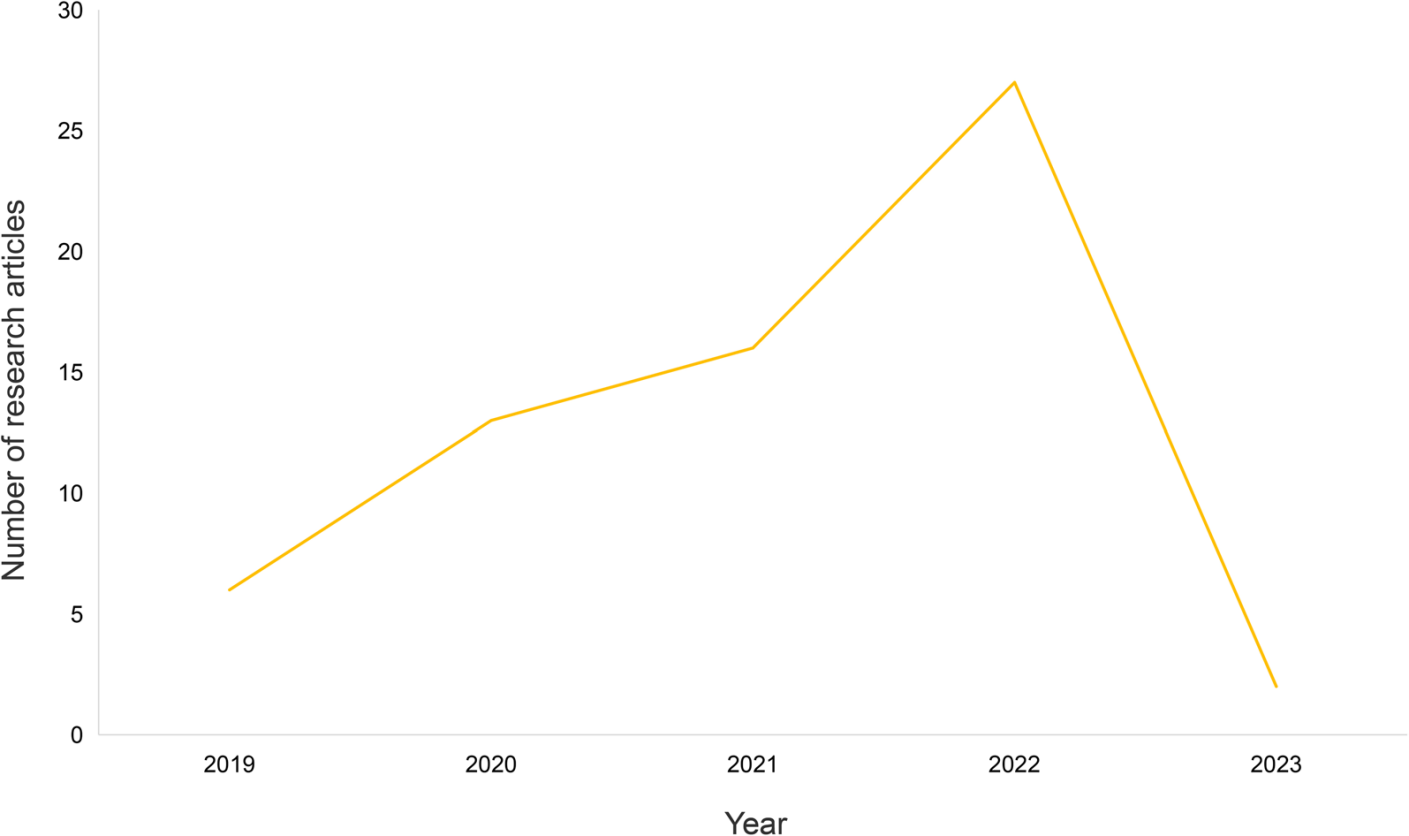
Total number of the research articles searching through PubMed, MEDLINE, Web of Science, Embase

**Fig. 3:**
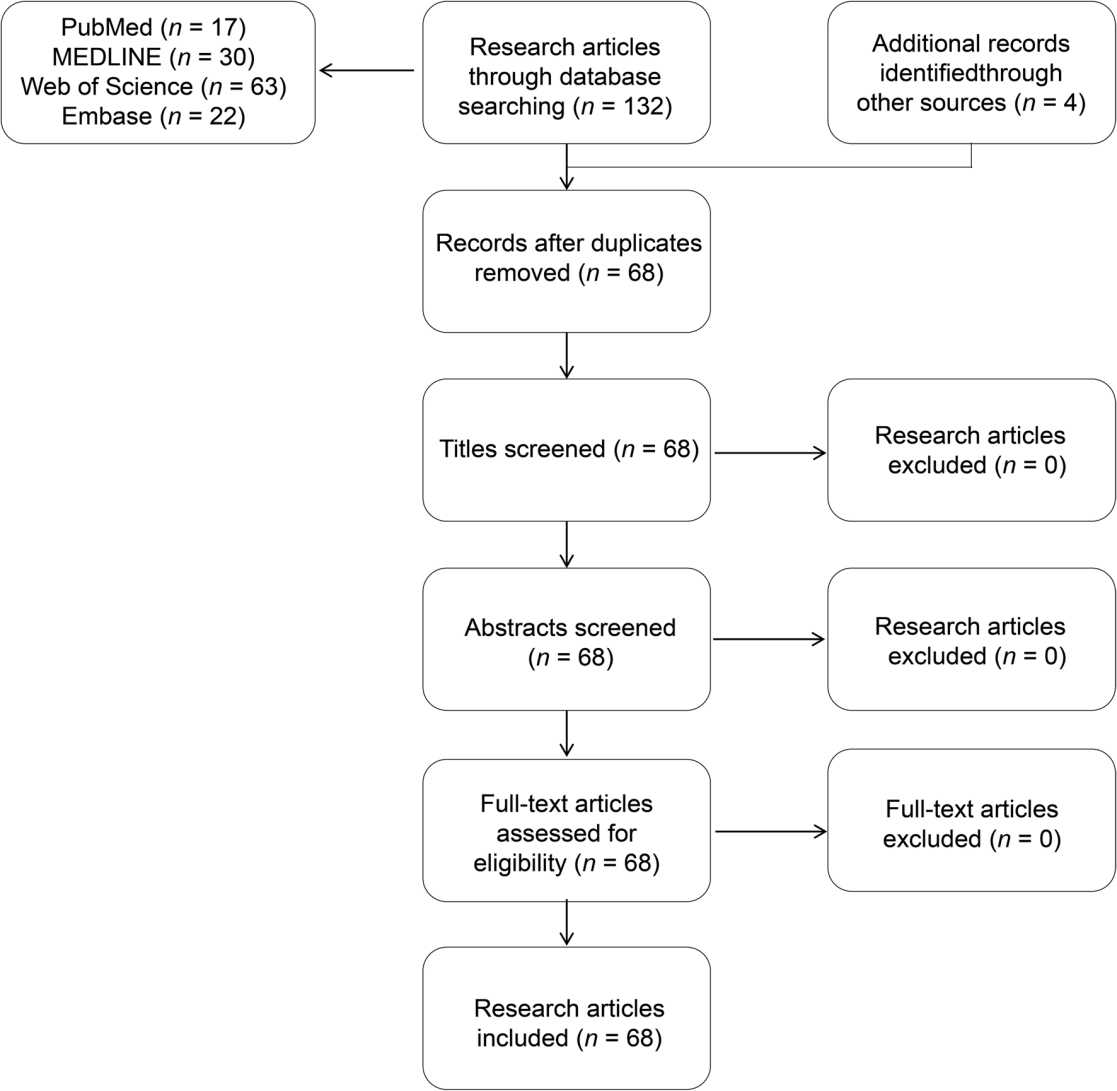
The flow diagram of the research articles selection process

A total of 64 research articles were found through searching in PubMed, MEDLINE, Web of Science, and Embase from 2019 to 2023, with the four fields fully listed in Fig. 2, showing the peak years for the research articles occurred after 2020, while the highest number of 27 being in 2022 [4, 10–72]. Search items most mentioned in the research articles’ titles were “development or progress”, “challenge”, “gap or disparity”, and “China and Chinese”, which is shown in Table 1. Additionally, we found four reports in the Institutional Repository for Information Sharing of WHO [7, 73–75]. The flow diagram of the research articles selection process is shown in Fig. 3.

**Table 1:** Results of the research articles searching through PubMed, MEDLINE, Web of Science, Embase by four fields restricted in title, abstract, keyword or topic

Year	Field: development or progress	Field: challenge	Field: China’s contribution	Field: gap or disparity
2019	4	2	0	0
2020	7	3	0	3
2021	12	3	1	0
2022	26	0	0	1
2023	1	1	0	0
Total	50	9	1	4

From the review of research articles and reports, we found that the malaria burden was high in many low-income Africa countries with little capacity to fund malaria control and eradication programs, the fight against malaria in these regions was likely to be hindered by COVID-19. Indeed, malaria interventions, such as SMC, have been affected during the pandemic. So gap analysis was needed to investigate the challenges and gaps of SMC since the outbreak of COVID-19.

### Gap analysis of SMC

#### Development of SMC in recent years

As children are at high risk of being infected with malaria, SMC needs to have an overview of the number of new malaria cases per 1000 high-risk groups in Africa. The reduction of disease burden in the region is shown in Table 2 during the period of 2009–2019. As of the end of 2021, SMC had been implemented in 13 countries in the Sahel region. There is no direct attribution of reduction due to SMC alone proven, but it is believed that SMC has indeed reduced the disease burden on children in the Sahel region. SMC has been proven to be safe and effective. A meta-analysis of six randomized controlled trials in children under five in West Africa found a protective efficacy against clinical malaria of 74% (95% *CI*: 62–83%, *P* < 0.001) [76]. Issiaka et al. conducted a retrospective study. It assessed the impact of SMC on hospitalizations and deaths of children under five years of age during the second year of implementation in the health district of Ouelessebougou in Mali. A total of 6638 children were surveyed, with 2759 children in the SMC intervention areas and 3879 children in the control areas. All-cause mortality rate per 1000 person-years was 8.29 in the control areas compared to 3.63 in the intervention areas. The incidence rate of all causes of hospital admissions was 19.60 per 1000 person-years in the intervention group compared to 33.45 per 1000 person-years in the control group. Implementing of SMC was associated with substantially reducing in hospital admissions and all-cause mortality [49].

**Table 2:** Disease burden index of 10 countries in the Sahel region in 2019 (by gender). Data source: World Malaria Report 2021

Year	Number of cases				Number of death		
	Point	Lower bound	Upper bound	% *Plasmodium vivax*	Point	Lower bound	Upper bound
2010	244,000	225,000	269,000	6.70%	698,000	650,000	764,000
2011	237,000	219,000	259,000	6.90%	651,000	611,000	703,000
2012	233,000	216,000	254,000	6.70%	614,000	578,000	664,000
2013	227,000	211,000	247,000	5.60%	589,000	553,000	640,000
2014	224,000	206,000	243,000	5.10%	569,000	532,000	620,000
2015	224,000	207,000	243,000	4.50%	562,000	524,000	619,000
2016	226,000	210,000	246,000	4.30%	566,000	527,000	627,000
2017	231,000	214,000	251,000	3.60%	574,000	537,000	643,000
2018	227,000	209,000	247,000	3.10%	588,000	521,000	633,000
2019	227,000	208,000	248,000	2.80%	588,000	521,000	642,000
2020	241,000	218,000	269,000	1.90%	627,000	583,000	765,000

**Fig. 4:**
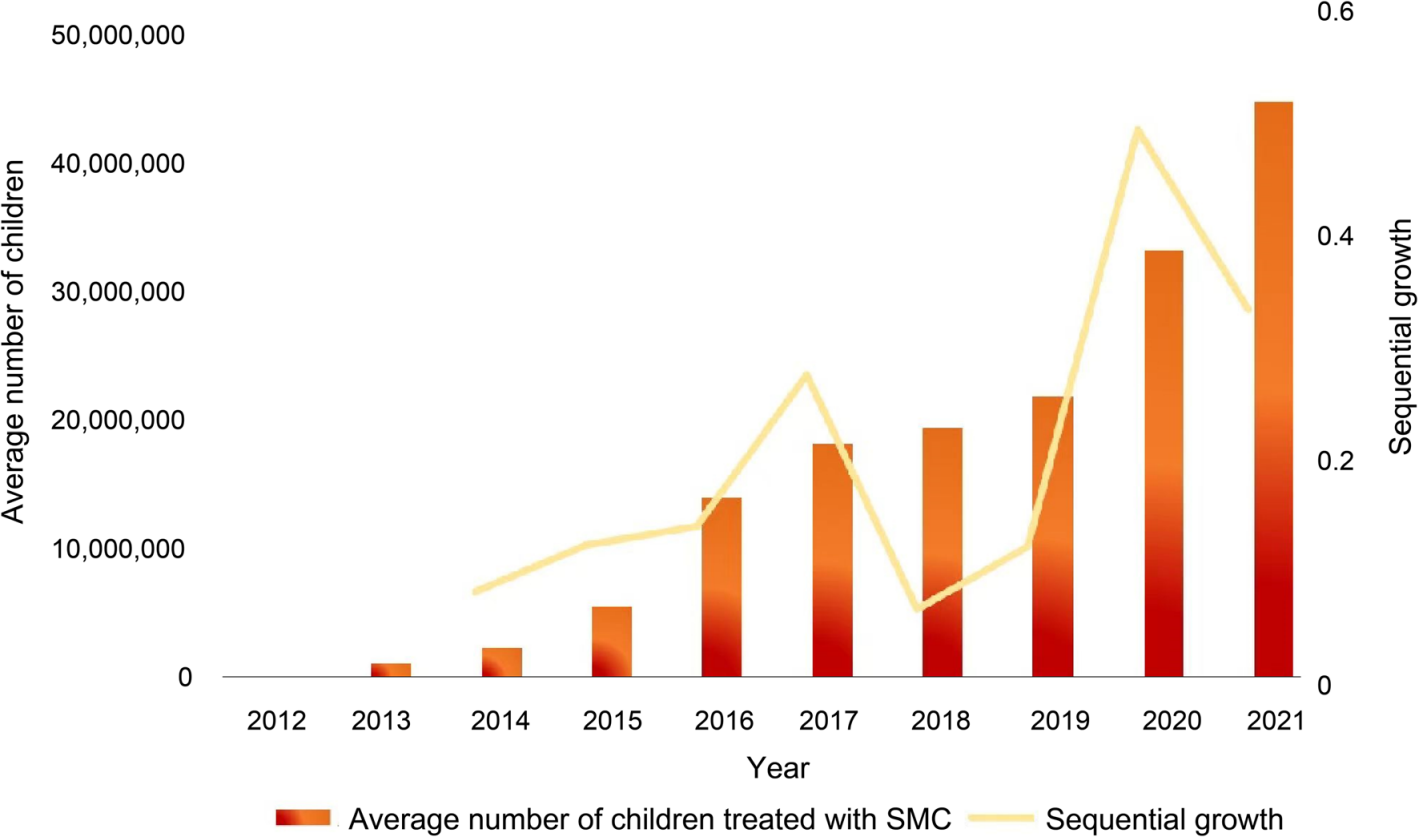
Average number of children treated with SMC per cycle, by year, in countries implementing SMC, 2012–2021. SMC: Seasonal Malaria Chemoprevention

Since the outbreak of COVID-19, major partners, such as the Global Fund, UNICEF, WHO, the World Bank, the Korea International Cooperation Agency, and the Bill & Melinda Gates Foundation in collaboration with local countries in the Sahel region, have made great efforts to maintain malaria treatment and prevention services. Despite the delays in the SMC campaign, 11.8 million children received SMC protection in 2020, showing steady growth in the number of children covered. More gratifying is that the average number of children treated per cycle of SMC increased from about 0.2 million in 2012 to almost 45 million in 2021. The total number of treatment doses delivered in the 15 countries implementing SMC in 2021 was about 180 million, as shown in Fig. 4 [5]. While the program is operationally complex and dependent on a range of moving parts, the outreach of the SMC program has been expanded at a relatively steady rate. The Malaria Consortium, for instance, has strengthened the supply chain and developed the use of digital tools to support implementation, and prioritize the available resources among SMC-implementing countries.

It is worth noting that WHO updated its recommendations on SMC in the WHO Guidelines for Malaria in June 2022, which is more flexible and different from the original 2021 version in a couple of significant ways: Areas outside the Sahel with highly seasonal malaria transmission could also benefit from SMC; SMC can be given without defining the specific number of cycles; SMC can be extended to children over six years old. The move by WHO has enabled National malaria programs (NMPs) in local countries to tailor their strategies to the settings and increase the impact of SMC when applied together with LLINs and the malaria vaccine [51, 60, 71].

In the past few years, the National Institute of Parasitic Disease of China CDC has held rounds of dialogues with WHO, the Global Fund, and Roll Back Malaria (RBM) to seek opportunities for alignment and synergy on SMC in terms of the supply of WHO-prequalified antimalaria drugs and LLINs to the Sahel region and sharing of China’s expertise. In the past years, China has expanded its engagement with global partners to facilitate the development of malaria programs at the national and regional levels in East Africa and the Greater Mekong Sub-region. The Sahel region has become another area where a new type of cooperation between China and related partners might be established through SMC [66, 70].

#### Challenges for SMC in the Sahel region

Despite the benefits of SMC in the fight against malaria, the disease remains a major public health problem in most countries implementing this strategy [77]. West African countries, including Burkina Faso, Mali, and Niger, remain heavily affected by the disease, with high prevalence and mortality rates [49]. Several studies have reported a high prevalence of asymptomatic malaria or a high incidence of hospital admission or death due to malaria in SMC areas [79, 80]. The challenges include but are not limited to (1) a shortage of fully covered monthly courses due to the lack of human, material, and financial resources; (2) lack of adherence to the second and third doses of amodiaquine; (3) four courses of SMC are not sufficient to cover the entire malaria transmission season in areas where the peak transmission lasts longer; (4) additional interventions beyond LLINs are needed to consolidate SMC efforts.

#### Gaps in supplying quality-assured and affordable health commodities

##### WHO-prequalified pediatric antimalaria drug

**Fig. 5:**
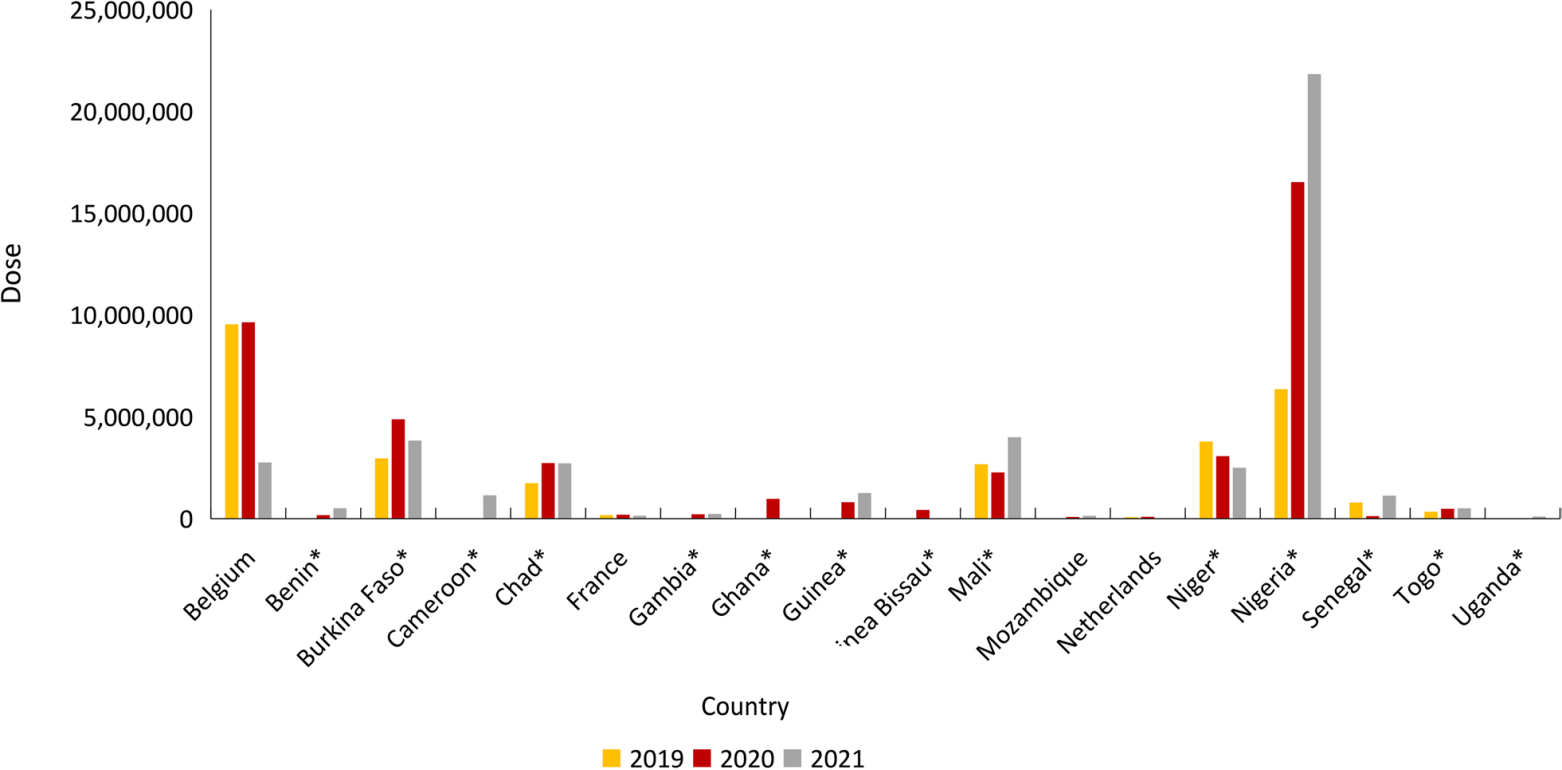
Histogram of export countries of SPAQ-CO of Guilin Pharma in 2019–2021; *Countries that SMC had been implemented. SPAQ-CO: Combined packaging of sulfadoxine-pyrimethamine and amodiaquine; SMC: Seasonal Malaria Chemoprevention

**Fig. 6:**
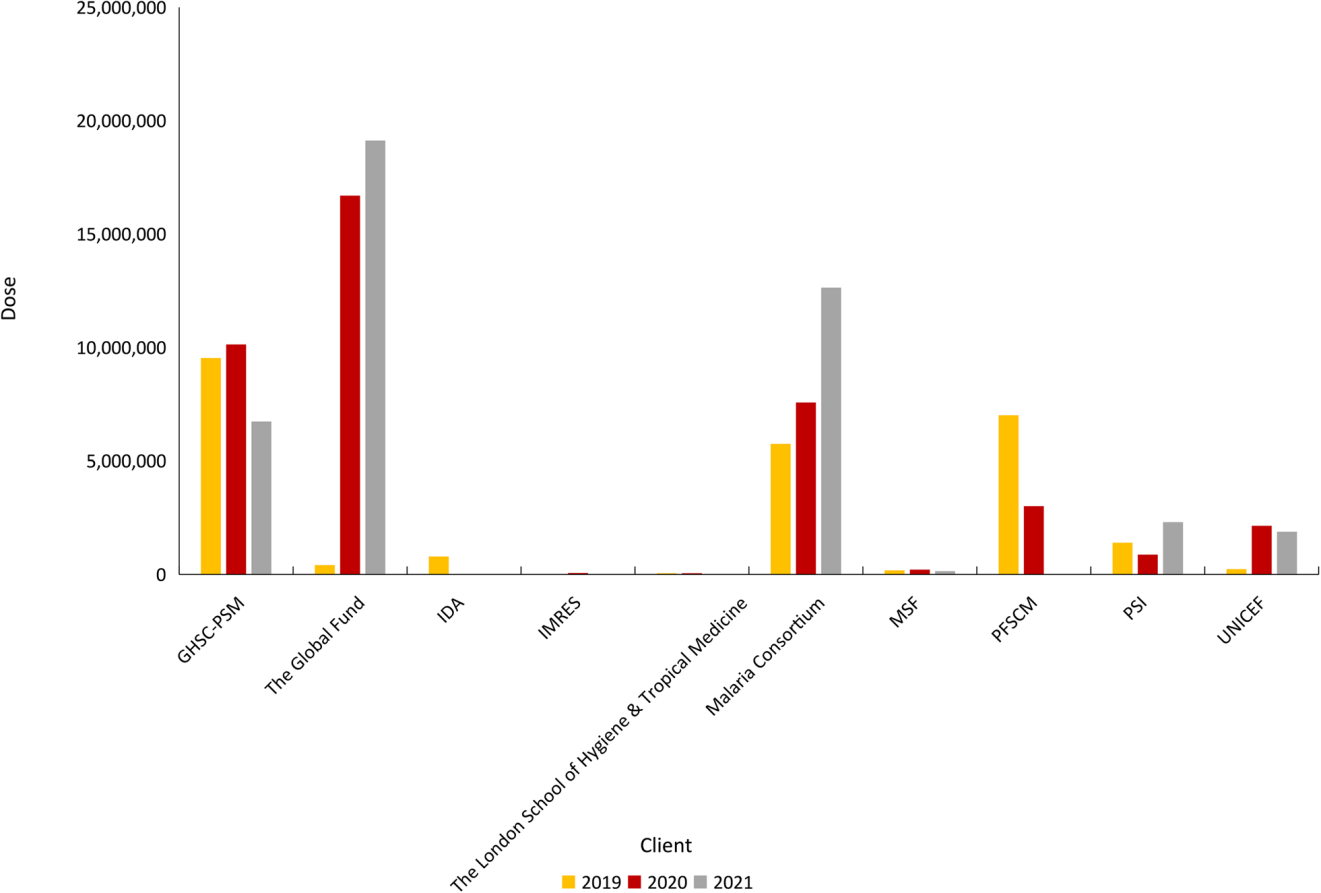
Histogram of export clients of SPAQ-CO of Guilin Pharma in 2019–2021; SPAQ-CO: Combined packaging of sulfadoxine-pyrimethamine and amodiaquine; GHSC-PSM: Global Health Supply Chain Program-Procurement and Supply Management; IDA: International Development Association; IMRES: IMRES B.V.; MSF: Médecins Sans Frontières; PFSCM: Partnership for Supply Chain Management; PSI: Population Service International; UNICEF: United Nations International Children's Emergency Fund

SPAQ-CO is administered monthly with the intake of amodiaquine once daily for three days and of sulfadoxine-pyrimethamine one day. The gap is that there are few companies producing WHO-prequalified pediatric antimalaria drug, such as SPAQ-CO. In China, Fosun Pharma is the supplier of antimalarials to the Global Fund, United Nations International Children's Emergency Fund, WHO, and African countries. Guilin Pharma is a branch company of Fosun Pharma, which has developed and produced dispersible SPAQ-CO for SMC. WHO prequalified dispersible SPAQ-CO in December 2018. In 2019, Guilin Pharma supplied more than 25 million doses of dispersible SPAQ-CO to 10 countries and nine clients. In 2020, Guilin Pharma supplied more than 40 million doses to 16 countries and nine clients. In 2021, Guilin Pharma supplied more than 40 million doses to 15 countries and six clients. Figure 5 shows the histogram of export countries of dispersible SPAQ-CO in the past 3 years. Figure 6 shows the histogram of export clients of dispersible SPAQ-CO in the past 3 years.

In the past 3 years, there were more than one hundred million children in the Sahel region with a high incidence of malaria benefiting from SMC and SPAQ-CO. Guilin Pharma has effectively helped decrease malaria's morbidity in children in the Sahel region and become the biggest supplier of SPAQ-CO. Besides Guilin Pharma, Skant Healthcare Ltd. and Macleods Pharmaceuticals Ltd. in India have also supplied a small amount of dispersible WHO-prequalified SPAQ-CO for pediatric use.

##### WHO-prequalified LLINs, including pyrethroid-PBO bed nets

Long lasting insecticidal mosquito nets (LLINs) are another effective tool against malaria recommended by WHO. To better prevent malaria, SPAQ-CO is usually used with LLINs, including the pyrethroid-PBO bed net. The pyrethroid-PBO bed net is suggested by WHO to be deployed in areas with ongoing malaria transmission where the principal malaria vectors exhibit pyrethroid resistance. PBO bed nets act by inhibiting certain metabolic enzymes, primarily oxidases, which are likely to provide more excellent protection than pyrethroid-only LLINs, where mosquitoes display mono-oxygenase-based insecticide resistance mechanisms [81]. WHO conducted surveys from 2009 to 2011 in 17 sub-Saharan African countries and reported that the median net usage rate was 91%, with an interquartile range of 82%-98% usage, and LLINs are assumed to last 2.11 years on average, consistent with the decay model.

Quite a number of companies produce WHO-prequalified LLINs, including pyrethroid bed nets, but not many are involved in producing pyrethroid-PBO bed nets. Despite the fact that Chinese companies are major suppliers of bed nets, but only a few Chinese companies are capable of manufacturing pyrethroid-PBO bed nets, such as Tianjin Yorkool Technology Group and De’an Sulilong Textile Co., LTD.

### China’s potential contribution to SMC based upon the key learnings from its long-term malaria elimination campaign

#### Potential for China to join the multilateral cooperation in SMC

**Fig. 7:**
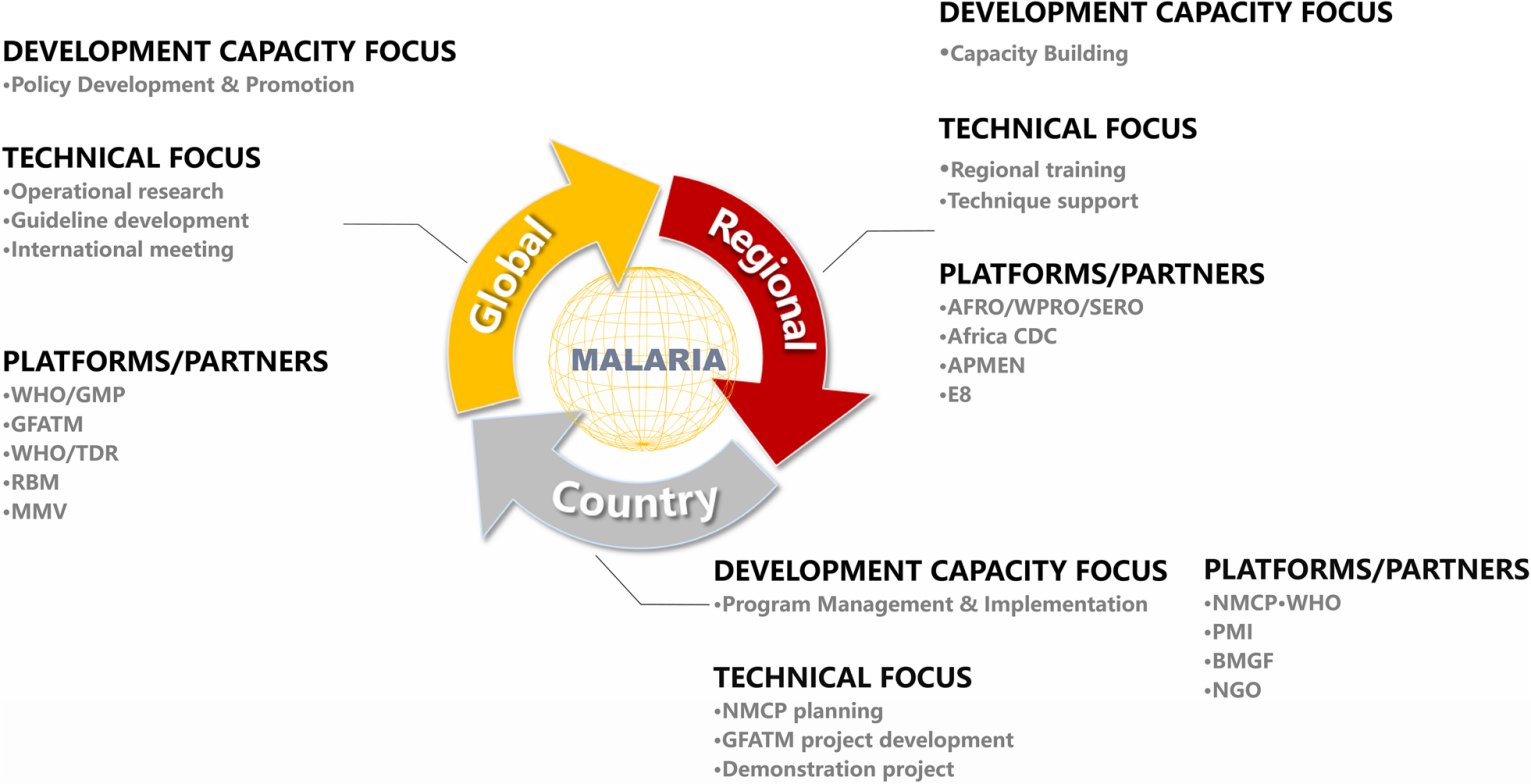
Strengthened China’s Capacity and Roles in Global Health through Malaria Elimination; AFRO/WPRO/SERO: Africa Regional Office/Western Pacific Regional Office/Southeast Regional Office; Africa CDC:Africa Centers for Disease Control; APMEN: Asia–Pacific Malaria Elimination Network; E8:Eight countries Eliminate malaria; NMCP: National Malaria Control Programme; WHO: World Health Organization; PMI: The President’s Malaria Initiative; BMGF: Bill Melinda Gates Foundation; NGO: Non-Governmental Organizations; GMP: Good Manufacturing Practice; GFATM: Global Fund to fight Aids Tuberculosis and Malaria; TDR: Tropical Disease Research; RBM: Roll Back Malaria; MMV: Medicines for Malaria Venture

China used to have a high burden of malaria, especially in the rural areas. As a country certified by WHO as malaria-free in June 2021, China has accumulated extensive experience in malaria control, driving down malaria incidence over the last decades to zero indigenous malaria cases. The launch of the Global Development Initiative (GDI) by China in 2021, together with the cooperative projects under the Belt and Road Initiative linking China with other parts of Asia, Africa, and Europe, has maximized the synergies for the implementation of key initiatives in the health sector and provide tremendous opportunities for the global health community, and in particular the fight against malaria in Africa. It is essential to strengthening further China’s capacity and role in global health through malaria elimination programs, especially in the three dimensions of country, regional, and global (Fig. 7).

#### Supply of quality-assured drugs and LLINs for SMC

China is a major supplier of pharmaceutical and vector control products against malaria worldwide [82]. The viable form of China’s participation in SMC cooperation is to provide more quality and affordable SPAQ-CO, bed nets, larvicides, etc., through multilateral or bilateral aid channels. Dispersible SPAQ-CO for pediatric use produced by Guilin Pharmaceutical has been the most widely used antimalarial for SMC in the Sahel region in recent years. According to Medicines for Malaria Venture (MMV), demand for SMC is likely to increase in the coming years due to the extension of the age range to include children up to 10 years of age and an increased duration of coverage (up to 5 months based on changing epidemiological patterns). Ensuring a steady supply of SPAQ will therefore be crucial [83]. Since SMC needs to maintain a high volume of drugs and LLINs every year, a sustained and large-scale supply from China, in the long run, would be critical to keeping SMC afloat. In addition, Chinese companies may also join forces to develop new treatments if the efficacy of SPAQ should be compromised by drug resistance.

#### Know-how transfer and experience sharing

China lowered its malaria burden from 30 million cases per year in the 1940s to zero indigenous cases in 2017. It was certified malaria-free by WHO on 30 June 2021. Although the malaria elimination process is different in each country because of different ecologies, epidemiology, health system context, and general socioeconomic circumstances, the key to success bears similarities in all settings [84]. China’s experience and expertise in malaria elimination can be shared with high-burden countries through know-how transfer, training and on-site guidance, operational research, and data analysis. The resources of Chinese experts in international anti-malaria work (anti-malaria poverty reduction management, case management, vector control, etc.) can be explored for promoting malaria control in the Sahel region. Considering the implementation of SMC, China-Africa antimalarial cooperation can be repositioned to maximize the integration of domestic and foreign investments, make full use of China’s experience, and support the malaria elimination process in the Sahel region.

Regarding malaria prevention and control experience, China’s experience has become a valuable reference for endemic countries. China’s 1-3-7 surveillance and response model, for instance, could apply to pilot studies, which has proved to be a key factor in reaching zero locally-transmitted malaria cases and maintaining the interruption of transmissions in the country. The 1-3-7 model requires that confirmed malaria cases are reported within one day, investigation of confirmed malaria cases are implemented within three days, and targeted control measures are adopted to prevent further transmission within seven days. Table 3 aims to explore the potential contribution to SMC based upon key learnings from China in mass drug administration [85].

**Table 3:** Potential contribution to SMC based upon key learnings from China

Problems to be solved	China’s malaria elimination practices
Incomplete coverage particularly in rural Sahel characterized by poor infrastructure and limited access to essential antimalarial products	***Practice 1*** Before the start of MDA every year, the provincial, county and township governments holds a mobilization meeting to sign a responsibility letter to agree to the implementation objectives and activities, and determines the number of people who need to take medicine according to the number of cases and targeted population, then provides the necessary drugs to the manufacturer in advance ***Practice 2*** The county governments holds a multisector meeting, including the county magistrate, health bureau, drug administration bureau, finance bureau, education bureau, radio and television bureau, local CDCs and heads of townships and health centers, to ensure the community’s adoption of medicine, and the whole participation rate should be > 85% as required ***Practice 3*** The medicine delivery team consisting of 3–5 members at least 1 doctor from the health center, 1 village cadre and 1 village doctor is organized from each natural village to send medicines from door to door ***Practice 4*** To improve targeted population compliance, the community empowerment campaign is conducted through publicity distributed via TV stations, radio stations, newspapers, etc.; knowledge on malaria control and prevention is posted on the outer walls of the village center and roadside houses in each natural village; a variety of health education products, i.e. aprons for home women, calendar pictures, magnetic soft screen doors, shopping bags, eye charts for students, etc. are distributed for the key stakeholders in the communities ***Practice 5*** Subsidies and foods (yoghurt, bread, eggs, etc.) are provided to each drug delivery person to ensure the enthusiasm of the grassroots staff
Systems to monitor adverse drug reactions are extremely fragile	***Practice 1*** Tour supervisory teams at the provincial level and local supervisory teams at the county level are organized to supervise the implementation of MDA in all targeted communities ***Practice 2*** Trainings are carried out at the provincial, prefectural and county levels before implementation, including identifying potential contraindications to medications and identifying and treating adverse drug reactions
Insufficient funding for SMC scale-up	***Practice 1*** The national-, provincial- and county-level governments disburse special funding for the purchase of medicines, personnel training and staff subsidies, respectively
Despite SMC, malaria remains a major health issue in rural Sahel	***Practice 1*** To increase the rounds of MDA and/or targeted prophylaxis in the hotspot areas where the highest transmission continues throughout the year, such as implementing the MDA and/or targeted prophylaxis in the spring (before first peak transmission) and summer (before the second peak transmission) in the high- transmission areas in the 1970s ***Practice 2*** Based on the local transmission levels to implement the local-tailored interventions, i.e. to carry out piloted interventions in 10 selected counties where the falciparum malaria parasites was still prevalent under routine interventions, and in 8 selected counties where vivax malaria parasites was stubborn ***Practice 3*** To adjust specific drugs of MDA and/or targeted prophylaxis according to local transmission vectors and parasite, such as adding the primaquine to vivax malaria parasites
Additional interventions beyond LLINs are needed to consolidate SMC efforts	***Practice 1*** To strengthen the IRS coverage and usage of LLINs, as well as community mobilization and participation in consolidating the MDA and/or targeted prophylaxis efforts

SMC: Seasonal Malaria Chemoprevention; MDA: Mass drug adminstration; LLINs: Long lasting insecticidal mosquito nets; CDC: Center for Disease Control and Prevention

#### Conducting feasibility study for scale-up

SMC has undergone tremendous changes in age, dose, frequency, delivery, and other aspects, laying the ground for a scale-up. The age group of SMC drugs is no longer limited to children under five years old [86]. SMC has also been proven effective in reducing the incidence rate of clinical malaria in children under ten years old. The current evidence has extended the use cycle of SMC. SMC 3–4 cycles are used every month in a shorter transmission environment, and they can be increased to 6 cycles in a more extended transmission season. Regarding drug delivery, door-to-door delivery can significantly improve the coverage of SMC [87]. It is desirable for local countries or international organizations to launch a joint feasibility study or scoping mission with China’s malaria experts on how to scale up SMC through the alignment and synergy of China’s bilateral aid with ongoing programs at both national and regional levels.

#### Potential for narrowing the financial gap

Despite the existing investments by governments and multilateral partnerships such as the Global Fund, there is still a funding gap of about 40.5 million USD to cover the total cost of seasonal malaria chemoprevention to prevent malaria among children [5]. China has the potential to contribute financial resources to sustain the long-term investments needed to eliminate malaria in high-burden countries. Over the years, an amicable relationship has developed between China and African countries in the health field. Since China sent its first medical team to Africa in 1963, about 20,000 medical professionals have been dispatched to 51 African countries. China-Africa health cooperation, including in the area of malaria has grown through sending medical teams, training programs, donations of medicines and medical equipment, joint research, and academic exchanges [88]. Co-investment or co-funding for SMC might be undertaken through tripartite cooperation between China, an international organization, and high-burden countries in the region. An alignment of China’s South-South Cooperation and Assistance Fund with the existing multilateral contributions by major partners on SMC could be achievable, through which financial resources can be more proportionally allocated to reach out to areas or populations still uncovered or partially covered.

### Implications

#### An integrated and coordinated approach is needed to boost investment in SMC

To ensure a sustained financial contribution to the scale-up of SMC, all major partners should work more closely: (1) to build a platform to pool investments and increase domestic financing by attracting and channeling additional funds from various stakeholders; (2) to promote blended financing by aligning strategic investment models between the public and private funders and complement the public investment; (3) to issue the social impact bond on SMC in countries where conditions are relatively mature; (4) to explore the debt swap scheme to convert debt repayments into targeted investments in SMC given the accumulated experiences on the part of the Global Fund. Based on past experiences, we need to realize that the purpose of SMC financing is not to expand the scale of funds without restriction, but to pursue cost-effective improvements to achieve the best results with the least amount of money [89].

#### More investigational tasks for SMC to fulfill

In contrast to World Malaria Report 2021, the 2022 update of WHO’s malaria chemoprevention tools are more flexible, adjusting the strategy to accommodate local needs. It no longer specifies geographical locations, allowing for more elasticity in recognizing age-based risk among children, transmission intensity thresholds, and numbers of cycles. The effectiveness of chemoprevention programs will be influenced by many contextual factors (e.g., the intensity of malaria transmission, degree of seasonal variation in transmission, age groups targeted by chemoprophylaxis programs, the preventive effect of drugs used, frequency of administration, duration of protection per regimen, availability of drugs, coverage obtained, adherence to recommended regimens), and the variety of interventions deployed in each setting. For example, due to varied summer cycles in different countries, the cycle of SMC can be extended by 5–6 cycles. Therefore, more data and evidence must be collected better to tailor SMC programs to local circumstances [67].

#### Beyond the Sahel region

SMCs in West and Central Africa are examples of how effective antimalarial drugs for intermittent treatment can be making a profound impact on malaria's incidence and mortality rates [47]. In eastern and southern Africa (Angola, Botswana, Ethiopia, Malawi, Tanzania, Malawi, Mozambique, Namibia, Zambia, and Zimbabwe), about 14.1 million eligible children are expected to benefit from SMC, including 9.6 million children under the age of 5 every year [5]. This projection makes it urgent to create alignment, synergy, and coordination on a larger scale. However, one of the main restrictions on expanding of SMC to other regions in sub-Saharan Africa is the resistance to one or two of the drugs. In addition, the region where SMC is currently deployed is threatened by the development of drug resistance. Therefore, it is essential to observe the prevalence of drug resistance and provide technical guidance for national malaria programs to adopt, adapt and implement WHO’s updated recommendations. In parallel, this challenge creates opportunities for innovation to find new antimalarial drug combinations suitable for chemical prevention in other regions where seasonal transmission also occurs and where the incidence rate of malaria is high, which will benefit from the same approach.

#### Robustness of SMC

Previous studies have shown that SMC combined with screening other diseases can have unexpectedly obvious effects. For example, in the second SMC cycle, SMC combined with malnutrition screening indicates that the probability of a patient's weight not reaching a healthy level standard is significantly reduced [58, 90]. Therefore, combining seasonal malaria chemical prevention with other interventions can increase coverage and reduce comorbidity. For instance, with the support of the Bill & Melinda Gates Foundation, the Malaria Consortium piloted the integration of vitamin A supplementation (VAS) with SMC campaigns in Bauchi state, Nigeria, from 2021 to 2022, and the co-implementation has facilitated VAS coverage and SMC implementation. In the years ahead, all stakeholders need to adopt a holistic and forward-looking approach. Integrating the VAS program into a community-led paradigm and mobilizing more human resources from the grassroots level are top priorities to be reckoned with in the years to come [62].

### Limitation

This article has several limitations. First, it is mainly focused on published peer-reviewed journal articles and therefore grey literature has been excluded. Second, the language restriction limits the pool of available studies and findings as most of the Sahel countries are francophone. Third, there is a lack of official and open source data to delineate China’s medical aid to Africa in the past decades.

## Conclusions

Through a decade of joint efforts, SMC has developed across the Sahel region and is poised to expand to more geographies, and cover more age groups among children during the malaria transmission season. Also, a combination of necessary preventive and curative activities may prove beneficial both for targeted populations and for health system strengthening in the long run.

More action is necessary to promote the partnership on SMC with the following recommendations:

First, China, a WHO-certified malaria-free country with accumulated demonstrated managerial and technical expertise and experience, can be a major contributor to SMC with various roles. The outcomes achieved so far through SMC should be attributed to established partnerships and networks that mobilize resources and facilitate its implementation. More space should be available for diverse customized approaches and methodologies and broader participation from governments, international organizations, civil society, the private sector, NGOs, philanthropic foundations, and academia [46].

Second, it is high time to explore China’s contribution to SMC, given its accumulated expertise in malaria elimination, commodity supply capability, and potential financial support. A clear road map should thus be drawn to get China, as well as other countries with relevant experience, involved in a way that is supplemental and beneficial to the existing partnerships. As more quality resources from various channels are pooled together, integrated, and synergized, SMC is sure to be a game changer in our journey to roll back malaria, which demonstrates how crucial it is to join forces to address common health challenges in low-resource settings.

## Data Availability

Not applicable.

## Abbreviations

Africa CDC: Africa Centers for Disease Control
AFRO/WPRO/SERO: Africa Regional Office/Western Pacific Regional Office/Southeast Regional Office
AIDS: Acquired immune deficiency syndrome
APMEN: Asia–Pacific Malaria Elimination Network
AQ: Amodiaquine
BMGF: Bill & Melinda Gates Foundation
CDC: Center for Disease Control and Prevention
COVID-19: Coronavirus disease 2019
E8: Eight countries eliminate malaria
GDI: Global Development Initiative
GFATM: Global Fund to fight Aids Tuberculosis and Malaria
GHSC-PSM: Global Health Supply Chain Program-Procurement and Supply Management
GMP: Good Manufacturing Practice
IDA: International Development Association
IMRES: IMRES B.V.
IPTsc: Intermittent preventive treatment of malaria in school-aged children
KOICA: Korean International Cooperation Agency
LLINs: Long lasting insecticidal mosquito nets
MDA: Mass drug administration
MMV: Medicines for Malaria Venture
MSF: Médecins Sans Frontières
NGOs: Non-Governmental Organizations
NMCP: National Malaria Control Programme
NMP: National Malaria Programme
PFSCM: Partnership for Supply Chain Management
PMI: The President’s Malaria Initiative
PMC: Perennial Malaria Chemoprevention
PSI: Population Service International
RBM: Roll Back Malaria
SMC: Seasonal Malaria Chemoprevention
SP: Sulfadoxine-pyrimethamine
SPAQ-CO: Combined packaging of sulfadoxine-pyrimethamine and amodiaquine
SSA: Sub-Saharan Africa
TDR: Tropical Disease Research
UNICEF: United Nations International Children’s Emergency Fund
VAS: Vitamin A supplementation
WHO: World Health Organization
